# Mid-Term Results of Left Carotid-Subclavian Bypass in Patients Undergoing Zone 2 TEVAR

**DOI:** 10.21470/1678-9741-2021-0597

**Published:** 2022

**Authors:** İlker İnce, Levent Altınay, Senan Huseynov, Melike Şenkal Zobu, Elif Şahin, Özgür Ersoy, İbrahim Duvan, Uğursay Kızıltepe

**Affiliations:** 1 SBU Dışkapı Yıldırım Beyazıt Training and Research Hospital, Ankara, Turkey

**Keywords:** Aortic Aneurysm, Thoracic Surgery, Carotid Arteries, Subclavian Artery, Low Extremity

## Abstract

**Introduction:**

The aim of this study was to present the mid-term results of patients who had undergone a carotid-subclavian bypass surgery after a thoracic endovascular aortic repair (TEVAR) stent-graft implantation with proximal landing at zone 2 of the aorta.

**Methods:**

A total of 66 patients had undergone TEVAR and carotid-subclavian bypass between January 2015 and May 2020 at our clinic. Five of these patients were lost to follow-up, so 61 patients were included in this retrospective study. At follow-up visits, patency of the carotid-subclavian bypass grafts was evaluated with physical examination and radiological imaging.

**Results:**

The mean follow-up time was 15.11±12.29 months (ranging from 1 to 56 months). There were 3 (4.91%) in-hospital deaths of patients admitted with bilateral lower limb and visceral malperfusion. There were also 2 (3.27%) deaths unrelated to the procedure. Carotid-subclavian graft occlusion occurred in 3 (4.91%) patients. The occlusion was detected with radiological imaging within a period of 12 to 24 months. The graft patency rate was 100% in the first 12 months. The mean graft patency time (survival) was 52.56±2.10 months.

**Conclusion:**

Periprocedural carotid-subclavian bypass surgery with synthetic grafts is a recommended procedure with high patency and acceptably low mortality and morbidity rates in TEVAR.

**Table:** 

Abbreviations, Acronyms & Symbols	
CSB	= Carotid-subclavian artery bypass
CTA	= Computed tomography angiography
ICU	= Intensive care unit
LSA	= Left subclavian artery
PICA	= Posterior inferior cerebellar artery
PTFE	= Polytetrafluoroethylene
RBC	= Red blood cells
SPSS	= Statistical Package for the Social Sciences
TEVAR	= Thoracic endovascular aortic repair

## INTRODUCTION

Carotid-subclavian artery bypass (CSB) is performed in peripheral occlusive disease of the subclavian artery and vertebrobasilar insufficiency, while protecting the patency of the ipsilateral hemodialysis access. Perioperative stroke and 5-year patency rates are 0-6% and 82-96%, respectively^[1-3]^. The indications for CSB are broadened following the introduction of thoracic endovascular aortic repair (TEVAR) in clinical practice^[4]^. Extension of the proximal landing zone up to the left carotid artery, thus covering the left subclavian artery (LSA) origin, increases the safety and efficiency of TEVAR in patients with an inadequate landing zone at zone 3. Revascularization of the subclavian artery reduces the risks of anterior/posterior circulation stroke as well as upper limb and spinal cord ischemia^[5]^. The aim of this study was to present the mid-term results of patients who had undergone a CSB surgery after a TEVAR stent-graft implantation with proximal landing at zone 2 of the aorta.

## METHODS

A total of 165 patients were treated with TEVAR, and 66 patients underwent TEVAR and CSB between January 2015 and May 2020 at our clinic. Five of these patients were lost to follow-up, so 61 patients were included in this retrospective study. Patients were followed up for 1-, 6-, 12-, and 24-month intervals. At follow-up visits, patency of the CSB grafts was evaluated with physical examination (by checking peripheral arterial pulses, looking for signs of limb ischemia, and comparing blood pressure measurements between the two arms) and radiological imaging (computed tomography angiography [CTA]). Pre- and postoperative chest radiograms were compared for diaphragm elevation to determine phrenic nerve palsy.

### Operative Technique

A 3-4-cm-long single skin incision was made from the lateral to the superomedial tips of the omoclavicular triangle. The internal jugular vein was exposed after dissection of the subcutaneous tissue. Then, the left common carotid artery was visualized and retracted with vascular tapes. The dissection was continued inferomedially over the clavicle to the anterior scalene muscle by mobilizing the pre-scalene fat pad upwards. The surgeon should be careful to avoid phrenic nerve injury at this stage of the dissection. Avoiding damage to the laterally located brachial plexus, the LSA was exposed at the lateral border of the anterior scalene muscle. Its division permits the proximal subclavian artery to be reached beyond the origin of the vertebral artery, which may allow ligation to preclude type II endoleak following TEVAR.

**Fig. 1:**
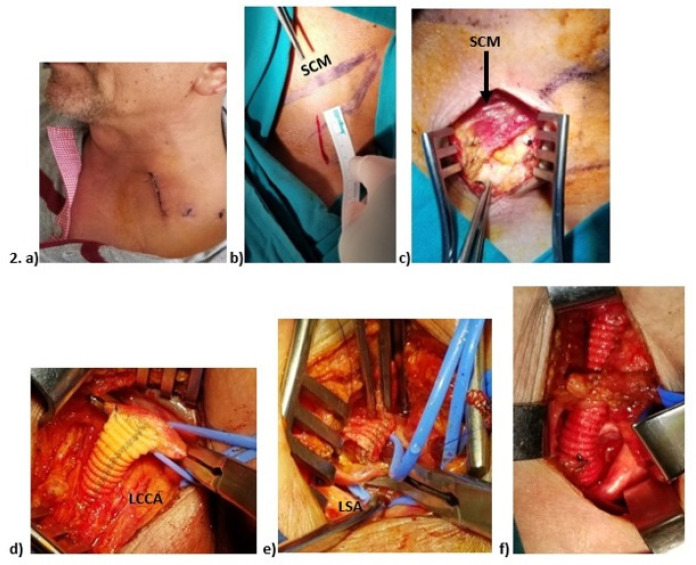
(A) A 3 to 4-cm-long skin incision was made in the supraclavicular region. (B, C) The sternocleidomastoid muscle was mapped on the skin and exposed. (D) Proximal anastomosis of the graft to the left common carotid artery. (E) Distal anastomosis of the graft to the left subclavian artery. (F) The bypass graft passing through the tunnel in the scalene fat pad. LCCA: left common carotid artery; LSA: left subclavian artery; SCM: sternocleidomastoid.

After administration of a proper dose of heparin, first the proximal end of a 7- or 8-mm Dacron (Polymaille C, Perous Medical, France) graft was anastomosed in an end-to-side fashion to the left common carotid artery. The graft body was threaded through a tunnel in the scalene fat pad and placed underneath it, and then the distal end of the graft was anastomosed to the left subclavian artery (Figure 1). The LSA was ligated with polytetrafluoroethylene (PTFE) tape when it was considered safe for the patient, whereas vascular plug implantation was performed if necessary due to a type II endoleak. The surgeon should keep in mind that ligation of the subclavian artery may result in catastrophic complications such as massive bleeding if the subclavian artery is fragile, as in patients with acute type B aortic dissections. We prefer endovascular occlusion of the subclavian artery with a vascular plug in case of a type II endoleak. Usually, the TEVAR procedure was completed in the same session as per routine.

### Statistical Analysis

The SPSS (Statistical Package for the Social Sciences) version 13 software was used for statistical analysis of the data. Qualitative data was expressed as percentages (%), and quantitative data as mean±standard deviation. The Kaplan-Meier curve was calculated for cumulative survival analysis.

## RESULTS

Patients’ demographic data are presented in Table 1. Mean follow-up time was 15.11±12.29 months (range 1 to 56 months). Three (4.91%) in-hospital deaths occurred in patients admitted with bilateral lower limb and visceral malperfusion. There were also 2 (3.27%) deaths unrelated to the procedure. One patient died of congestive heart failure 18 months after the operation, and 1 patient died of low cardiac output syndrome after emergency coronary artery bypass surgery, performed one month after the initial operation.

**Table 1:** Demographic data.

	N=61
**Age, mean±SD**	57.43±13.83
**Male gender, n (%)**	48 (78.68)
**Hypertension, n (%)**	34 (55.71)
**Hyperlipidemia, n (%)**	30 (49.23)
**Diabetes, n (%)**	36 (59.01)
**Cerebrovascular accident, n (%)**	1 (1.63)

SD=standard deviation

**Fig. 2:**
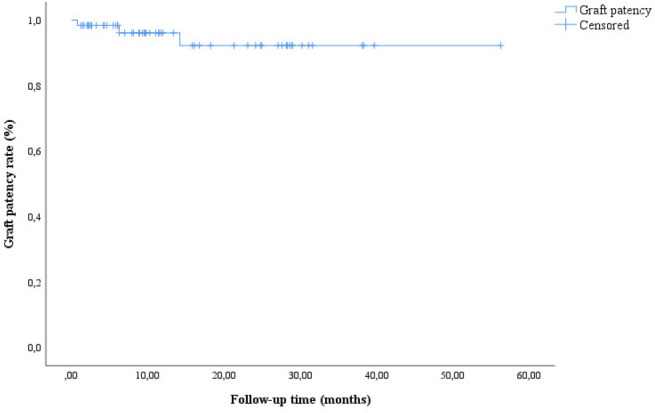
Survival curve related to left carotid-subclavian bypass graft patency.

CSB graft occlusion occurred in 3 (4.91%) patients. The occlusion was detected with radiological imaging within a period of 12 to 24 months. The graft patency rate was 100% in the first 12 months. There were no signs of vertebrobasilar insufficiency or other clinical complications in these patients, therefore, a secondary revascularization was not planned. The mean graft patency time (survival) was 52.56±2.10 months (Figure 2). Lymphorrhea occurred in 6 (10.16%) patients, and 1 of these patients also had chylothorax (1.6%). Revision surgery was needed in 1 (1.6%) of these patients because of a wound infection. The wound infection resolved, and no subsequent problems occurred in this patient. No graft infection occurred in the patient group of this study. Eight (13.11%) patients underwent revision surgery for bleeding. None of the patients had phrenic nerve palsy. Stroke due to contralateral carotid artery disease occurred in 1 (1.6%) patient. The mean number of packed red blood cells (RBC) units was 1.18±1.07 (ranged from 0 to 3). The mean length of stay in the intensive care unit (ICU) was 2.08±2.18 days (ranged from 1 to 14 days) and the mean length of hospital stay was 4.43±5.23 days (ranged from 1 to 26 days). Postoperative data are presented in Table 2.

**Table 2:** Postoperative data.

	N=61
**Emergent surgery, n (%)**	30 (49.18)
**Graft types, n (%)**	
6 mm-Dacron	2 (3.27)
7 mm-Dacron	34 (55.73)
8 mm-Dacron	22 (36.06)
9 mm-Dacron	1 (1.64)
8 mm-ringed PTFE	1 (1.64)
7 mm-non-ringed PTFE	1 (1.64)
**Graft occlusion, n (%)**	3 (4.91)
**Follow-up time, mean±SD (months)**	15.11±12.29
**Graft patency time, mean±SD (months)**	52.56±2.10
**Chylothorax, n (%)**	1 (1.63)
**Phrenic nerve palsy, n (%)**	0
**Stroke, n (%)**	1 (1.63)
**Lymphorrhea, n (%)**	6 (10.16)
**Total RBC units, mean±SD**	1.18±1.07
**Revision for bleeding, n (%)**	8 (13.11)
**Wound infection, n (%)**	1 (1.63)
**ICU stay, mean±SD (days)**	2.08±2.18
**Hospital stay, mean±SD (days)**	4.43±5.23
**Mortality, n (%)**	3 (4.91)

ICU=intensive care unit; RBC=red blood cells; SD=standard deviation

## DISCUSSION

The findings of this study showed that CSB surgery using synthetic grafts along with zone 2 TEVAR has high rates of early and late patency, as well as acceptable mortality and morbidity rates.

Blood flow to the LSA should be preserved to prevent posterior cerebral and spinal cord ischemia. The main blood supply to the spinal cord originates from the vertebral, segmental, and hypogastric arteries. The vertebral artery may be anatomically complete, partially duplicated, or asymmetric due to unilateral hypoplasia, or it may terminate in the posterior inferior cerebellar artery (PICA)^[6]^. The prevalence of PICA variation is about 4-5%^[7]^. Thus, losing the PICA blood supply causes a high risk of vertebrobasilar ischemic stroke (6.4%)^[8]^. Therefore, we performed LSA revascularization with open surgery (LSA-CSB bypass) or endovascular techniques (chimney or periscope) or by placing a physician-modified fenestrated TEVAR graft to protect the LSA blood flow in all our TEVAR patients, either elective or urgent, if the patient was eligible. Moreover, distal extensions of TEVAR grafts or additional TEVAR grafting into the descending aorta will endanger the spinal cord circulation, so prior LSA revascularization will also be useful in these situations.

In recent years, the TEVAR procedure has been a frequent CSB indication, and routine preoperative LSA revascularization is suggested in the Society for Vascular Surgery Clinical Practice Guidelines when the LSA is covered during non-urgent TEVAR. It is also strongly recommended if the collateral circulation may be compromised^[4]^.

The overall stroke rate after TEVAR was reported as 2.9% in a recent study^[9]^.The etiology is multifactorial. The coverage of important vessels with the device for aortic disease involving the arch vessels is one of the causes. It has been shown that >60% of patients have a dominant left vertebral artery with the contralateral vertebral artery rudimentary or absent, thus increasing the risk of stroke in case of unknowingly covering the LSA. Some studies have shown that intentional LSA coverage without revascularization has a higher overall (9.1-13% *vs.* 2-2.2%) and posterior stroke rates (5.1-5.5% *vs.* 2%), compared to LSA revascularization^[10-13]^. By contrast, Hajibandeh et al.^[14]^ reported in their more recent meta-analysis that LSA revascularization was not found to significantly reduce stroke rates after TEVAR procedures covering the LSA origin. We performed LSA revascularization in all our patients, either elective or urgent, as a routine.

The 5-year patency rates of CSB with prosthetic material was reported as 98%, and the same rate with venous grafts as 58%^[15,16]^. Law et al.^[17]^ compared the patency rates of all graft types used in CSB and reported that PTFE grafts had the best 5-year patency rate, which was 95.2±4.6%. Dacron grafts had the second best patency rate, 83.9±10.5%. They reported that saphenous vein grafts had the worst 5-year patency rate, 64.8±16.5%. However, they could not find any statistical significance between these values because of the high overall patency rate (*P*=0.200). Voigt et al.^[18]^, who utilized a PTFE graft in the majority (95.5%) of their patient group, reported 5-year synthetic graft patency rates of 97% in their study. In this study, a PTFE graft was used in 2 patients (3.2%) for CSB, and a Dacron graft was used in the remaining patients (96.8%). Dacron grafts were the only type of graft available at the time of this study. Küçüker et al.^[19]^ reported graft patency rates of 93.75% in 30 days and 100% in 18 months in their study, in which they had used Dacron grafts for carotid-subclavian bypass. Graft occlusions occurred in 3 patients with Dacron grafts, and the synthetic graft patency rate (95.1%) agreed with values reported in the literature.

The main surgical approach to arteries for a CSB surgery is the supraclavicular approach^[1]^. A transverse skin incision of approximately 8-10 cm-long, about 2 cm above the clavicle, starting from the clavicular head and extending to the lateral portion of the supraclavicular region, was described^[20]^. The medial approach includes division of both bellies of the sternocleidomastoid muscle, and the lateral approach includes division of only its lateral belly^[21]^. We made a much shorter incision in the omoclavicular triangle, preserving the sternocleidomastoid muscle and the scalene fat pad. We believe that this approach facilitates the postoperative wound healing process.

The scalene fat pad is divided in the common CSB technique in the literature^[18]^. We keep the scalene fat pad undivided, but we mobilize it. In our technique, it is dissected from the clavicle along the inferior border and elevated upwards. Then, we form a tunnel in the fat pad and performed the CSB through this tunnel. We believe that, by preserving the scalene fat pad, we protect the lymphatic tissue and reduce the likelihood of complications such as lymphorrhea due to lymphatic damage. Moreover, the synthetic graft body is placed underneath the scalene fat pad in our technique. We believe this will keep the synthetic graft away from the subcutaneous tissue and prevent future surgical site infections.

Delafontaine et al.^[22]^ reported higher rates of pulmonary complications and stroke in patients undergoing LSA revascularization with open bypass *versus* stenting (31.4 and 9.6% *vs.* 23.9 and 4.7%, respectively). They also reported a higher rate of left arm ischemia in patients with stented LSA revascularization *versus* open bypass revascularization (13.4% *vs.* 8.1%). These results contradict those of our study. Herein, we represent the results of open bypass LSA revascularization, and we did not have left arm ischemia in our patient group. We also have the results of endovascular LSA revascularization, which is scheduled for future publication.

### Limitations of the Study

Flow rates in the carotid and subclavian arteries were not measured with Doppler ultrasonography, as nearly half of the patients had undergone operations with urgent status. The preoperative patency of the carotid and subclavian arteries was evaluated only visually with CTA. We performed endovascular revascularization techniques such as chimney TEVAR or surgeon-modified fenestrated TEVAR for LSA revascularization instead of open CSB surgery in urgent patients or in patients who were not eligible for general anesthesia.

## CONCLUSION

Periprocedural CSB surgery with synthetic grafts can be performed with high patency rates and acceptably low mortality and morbidity rates in TEVAR procedures.
